# Analyzing foot care practices and diabetes management: a cross-sectional KAP study in a tertiary care hospital

**DOI:** 10.3389/fendo.2025.1547366

**Published:** 2025-05-29

**Authors:** Asma Malawi Alshahrani, Rajalakshimi Vasudevan, Afaf Aldahish, Yahia Alghazwani, Eman Shorog, N. Manusri, Rajeshri Dhurke, Puvvada Ranadheer Chowdary, Praveen Devanandan

**Affiliations:** ^1^ Department of Clinical Pharmacy, College of Pharmacy, Shaqra University (SU), Dawadimi, Saudi Arabia; ^2^ Department of Pharmacology, College of Pharmacy, King Khalid University, Abha, Saudi Arabia; ^3^ Department of Clinical Pharmacy, College of Pharmacy, King Khalid University, Abha, Saudi Arabia; ^4^ Department of Pharmaceutics, St. Peter’s Institute of Pharmaceutical Sciences, Hanamkonda, Telangana, India; ^5^ Department of Pharmacy Practice, St. Peter’s Institute of Pharmaceutical Sciences, Hanamkonda, Telangana, India

**Keywords:** diabetes mellitus, foot complications, knowledge, patients, educational practices

## Abstract

**Introduction:**

Diabetic foot complications, including foot ulcers and amputations, are significant contributors to morbidity and healthcare costs worldwide. Effective prevention requires comprehensive knowledge, positive attitudes, and consistent practices regarding foot care among diabetes patients. This study evaluates the Knowledge, Attitudes, and Practices (KAP) of diabetic patients in a tertiary care hospital setting to identify gaps and inform targeted interventions.

**Methodology:**

A cross-sectional study was conducted among 704 diabetes patients between January 2024 to September 2024 in a tertiary care hospital. A validated 20-item questionnaire assessed sociodemographic details, knowledge, attitudes, and practices regarding foot care. Descriptive statistics and logistic regression analysis were performed using SPSS to identify predictors of good foot care practices.

**Results:**

The study revealed moderate knowledge levels (mean score: 6.5 ± 1.8), with 54.1% of participants recognizing the importance of daily foot inspections. Positive attitudes were seen in 75.6%, but only 60.8% reported regular foot inspections, and 27% consulted healthcare professionals for foot care. Education level significantly influenced KAP outcomes, with participants having higher education showing better scores (p < 0.001). Longer diabetes duration (>10 years) was associated with improved practices (OR: 1.9; p = 0.01).The findings highlight critical gaps in knowledge and practice, especially among less-educated and newly diagnosed patients. Educational interventions and improved access to podiatric care are necessary to address these disparities. Strategies like community-based programs and telemedicine can enhance awareness and adoption of preventive measures, particularly in underserved areas.

**Conclusion:**

Enhancing foot care knowledge and practices among diabetes patients is vital to reducing the burden of diabetic foot complications. Targeted education and access to healthcare services are essential components of comprehensive diabetes management.

## Introduction

Diabetes mellitus (DM) is a chronic metabolic disorder characterized by elevated blood glucose levels due to either a deficiency in insulin production (Type 1 DM), or inadequate insulin production combined with insulin resistance (Type 2 DM) (1). According to the International Diabetes Federation (IDF), approximately 537 million people globally were living with diabetes in 2021, a number projected to rise to 783 million by 2045 (2). This surge has established DM as a significant public health concern, contributing to increased morbidity, mortality, and economic burden (3).

Among the most severe and costly complications of diabetes are diabetic foot problems, which include ulcers, infections, and lower-limb amputations (4, 5). These complications typically result from peripheral neuropathy, peripheral vascular disease, or a combination of both (6). Diabetic foot ulcers (DFUs) affect approximately 19–34% of individuals with diabetes over their lifetime and account for more than 50% of all diabetes-related hospital admissions. Furthermore, DFUs are the leading cause of non-traumatic lower-limb amputations worldwide (7, 8).

The importance of preventive foot care is well established. It relies heavily on patients’ knowledge, attitudes, and practices (KAP) to minimize complications. Studies have shown that patients with good foot care knowledge are more likely to engage in preventive behaviors such as daily foot inspections, proper footwear use, and regular consultation with healthcare professionals (9, 10). Crawford et al. highlighted the predictive value of proper education in preventing foot ulcers (9), while Lavery et al. emphasized the neglected opportunities in foot prevention among high-risk patients (10). Monteiro-Soares et al. also identified education as a key factor in diabetic foot care (11). Lavery et al. further noted risk factors for foot infections in diabetic patients (12). The International Diabetes Federation has also acknowledged the need for global focus on foot care (13).

A study from Saudi Arabia by Al Khowailed et al. demonstrated a significant association between educational level and proper foot care practices (14), echoing findings from other regions, including South Asia and Sub-Saharan Africa (15). The European Diabetes Policy Group has also emphasized structured diabetic foot care as part of comprehensive diabetes management programs (16).

Despite global efforts such as the St. Vincent Declaration and national diabetes programs (13), gaps remain in the implementation of effective foot care strategies, particularly in resource-limited settings. These disparities underscore the need for localized and context-specific research to inform targeted interventions.

This study aims to evaluate the knowledge, attitudes, and practices related to diabetic foot care among patients attending a tertiary care hospital. By analyzing the relationship between KAP scores and demographic variables, the study seeks to identify key gaps and provide actionable insights for developing tailored educational programs and preventive strategies that align with integrated diabetes care models.

## Methodology

### Study design

A cross-sectional study design was employed to evaluate the Knowledge, Attitude, and Practice (KAP) regarding foot care among patients with diabetes mellitus. The study aimed to identify gaps in awareness and behaviors that could contribute to better managing and preventing diabetic foot complications. This approach facilitated the collection of detailed data at a single point in time, allowing for the analysis of key factors influencing foot care practices.Ethical clearance for this study was obtained from the Institutional Ethics Committee of St. Peter’s Institute of Pharmaceutical Sciences (Approval No. St.Peter’s/IEC/2024/IV/11). Participants provided informed consent before completing the survey. Participation was entirely voluntary, with no incentives or coercion. The ethical principles of anonymity and confidentiality were strictly maintained.

### Study setting and population

The study was conducted at a tertiary care hospital in an urban setting, catering to a diverse population of patients with diabetes. A total of 704 adult patients with diabetes mellitus were recruited for the study. The study included adult patients (aged ≥18 years) diagnosed with either Type 1 or Type 2 diabetes mellitus for at least one year. Patients with gestational diabetes were excluded due to the temporary nature and different management of the condition. Additionally, individuals with active diabetic foot ulcers, infections, or those who had undergone lower-limb amputations were excluded, as the study focused on preventive foot care knowledge and practices rather than management of existing complications. Patients with severe cognitive impairments or those unable to provide informed consent were also excluded to ensure data reliability. This clearly defined set of criteria ensured a well-characterized study population appropriate for assessing foot care-related knowledge, attitudes, and behaviors. Participants represented a mix of socioeconomic and educational backgrounds, providing a broad understanding of foot care practices in this population.

### Sampling and data collection

Convenience sampling was used to recruit participants during their routine visits to the diabetes outpatient clinic. The entire study was carried out between January 2024 to September 2024, ensuring sufficient time to gather a representative sample. Each participant provided written informed consent before participating in the study. Data were collected using face-to-face interviews conducted by trained researchers, ensuring clarity of questions and completeness of responses.

### Questionnaire development

The questionnaire used in this study was a structured, prevalidated tool consisting of 20 items divided into three main domains: Knowledge (10 items), Attitude (5 items), and Practice (5 items). It was developed based on an extensive review of existing literature and diabetic foot care guidelines, with inputs from endocrinologists, podiatrists, and public health experts.

The Knowledge section included 10 multiple-choice and dichotomous (Yes/No or True/False) items that assessed participants’ understanding of various aspects of diabetic foot care. These included common signs of foot complications (e.g., swelling, redness, or sores), the importance of daily foot inspections, appropriate footwear use, the role of moisturizing and foot hygiene, frequency and necessity of professional foot checkups, and the risks associated with untreated ulcers, smoking, and poor blood glucose control. Each correct answer was awarded one point, resulting in a maximum possible knowledge score of 10.

The Attitude section contained 5 items measured using a 5-point Likert scale, ranging from “Strongly Disagree” to “Strongly Agree.” These items evaluated participants’ beliefs and motivation regarding foot care. Specifically, they explored perceived importance of foot care in diabetes management, confidence in identifying foot problems, belief in the benefits of professional care, willingness to adopt preventive measures, and sense of personal responsibility in maintaining foot health. Higher scores reflected more positive attitudes toward diabetic foot care.

The Practice section assessed the frequency and consistency of foot care behaviors through 5 items. These covered daily foot inspections, use of moisturizing lotions or creams, usage of specialized footwear (such as padded or custom-made shoes), routine foot hygiene (including washing and drying between toes), and frequency of visits to healthcare professionals for foot care. Responses were provided as Yes/No or using frequency-based categories (e.g., “Daily,” “Sometimes,” “Never”), and were scored based on their adherence to recommended practices.

The initial draft of the questionnaire was pilot-tested on a group of 50 diabetic patients to ensure clarity, cultural relevance, and ease of understanding. Based on the feedback received, minor modifications were made to improve clarity. Internal consistency of the final tool was confirmed with a Cronbach’s alpha value of 0.86, indicating good reliability.

### Data analysis

All data were entered into Microsoft Excel and analyzed using SPSS v26.0. Descriptive statistics were utilized to summarize demographic characteristics and KAP scores, expressed as frequencies and proportions n(%).

Knowledge scores were calculated by awarding one point for each correct answer, with a total possible score of 10. Attitude and practice responses were similarly scored based on predefined criteria, allowing for the categorization of participants into high and low scorers for each domain.

Inferential statistics were applied to identify significant associations between demographic variables and KAP outcomes: Chi-square tests were used to examine associations between categorical variables, such as education level and knowledge scores. Logistic regression was employed to determine predictors of good foot care practices, with variables such as knowledge, attitude, and diabetes duration included in the model. Pearson correlation was used to assess the relationship between knowledge and attitude scores. One-way ANOVA was applied to compare practice scores across different education levels.

A p-value <0.05 was considered statistically significant for all tests, ensuring robust conclusions. Results were presented in detailed tables and supported by narrative explanations for clarity. Ethical clearance was obtained prior to the study, and participants’ confidentiality was strictly maintained throughout the research process.

## Results

The demographic characteristics of the 704 participants are presented in **Table 1**. Of these, 396 (56.3%) were male and 308 (43.7%) were female. The majority of participants were between the ages of 40–60 years (59.1%), followed by those over 60 years (22.2%) and those under 40 years (18.8%).

**Table 1:** Demographic details.

Variable	n (%)
Gender	
Male	396 (56.3%)
Female	308 (43.7%)
Age Group	
<40 years	132 (18.8%)
40–60 years	416 (59.1%)
>60 years	156 (22.2%)
Education Level	
No formal education	98 (13.9%)
Primary education	204 (29.0%)
Secondary education	266 (37.8%)
Higher education	136 (19.3%)
Duration of Diabetes	
<5 years	286 (40.6%)
5–10 years	262 (37.2%)
>10 years	156 (22.2%)

In terms of education, 13.9% of participants had no formal education, while 29% had primary education, 37.8% had secondary education, and 19.3% had attained higher education. Regarding the duration of diabetes, 40.6% of participants had diabetes for less than 5 years, 37.2% between 5–10 years, and 22.2% for more than 10 years.

The knowledge levels of participants are summarized in **Table 2**. The mean knowledge score was 6.5 ± 1.8, indicating a moderate level of understanding regarding diabetic foot care. Among the key findings, 78.1% of participants correctly identified signs of diabetic foot complications, and 69.9% recognized the importance of using padded footwear. However, only 44.3% were aware of the need to consult a podiatrist, and just 37.9% correctly identified the recommended frequency for professional foot examinations. Awareness about the role of smoking as a risk factor was also low at 42%.

**Table 2:** Knowledge section.

Question	Correct responses, n(%)
Signs of diabetic foot complications	550 (78.1%)
Daily foot inspections	381 (54.1%)
Importance of padded shoes	492 (69.9%)
Diabetes-related nerve damage	503 (71.4%)
Role of moisturizing	358 (50.9%)
Consulting a podiatrist	312 (44.3%)
Blood sugar control prevents complications	566 (80.4%)
Frequency of professional foot examinations	267 (37.9%)
Risk of untreated foot ulcers	422 (59.9%)
Smoking as a risk factor	296 (42.0%)

Participants showed strong understanding in areas like the role of blood sugar control in preventing complications (80.4%) and the impact of diabetes-related nerve damage (71.4%). Nevertheless, the overall scores suggest specific gaps in awareness, particularly regarding professional care and lifestyle-related risk factors.

Attitudinal responses are detailed in **Table 3**. A substantial proportion of participants (75.6%) acknowledged the importance of foot care in diabetes management, and 70% supported the use of specialized footwear. Notably, 81.8% of respondents expressed motivation to adopt preventive foot care practices.

**Table 3:** Attitude section.

Statement	Positive responses, n(%)
Foot care is vital for diabetes management	532 (75.6%)
Specialized footwear is necessary	493 (70.0%)
Confidence in identifying foot complications	252 (35.8%)
Consulting healthcare professionals is worthwhile	408 (58.0%)
Motivation to adopt preventive measures	576 (81.8%)

However, only 35.8% reported confidence in their ability to identify foot complications, and 58% felt that consulting healthcare professionals was worthwhile. These findings reflect a generally positive attitude towards foot care but indicate a lack of confidence in personal assessment skills.

**Table 4** presents the reported foot care practices. While 60.8% of participants conducted daily foot inspections, only 27% reported regular consultations with healthcare providers for foot-related issues. Use of moisturizer was reported by 39.5%, and only 29% used specialized footwear consistently. On the other hand, 75.6% maintained regular foot hygiene, suggesting stronger adherence to basic care routines.

**Table 4:** Practice section.

Practice	n(%)
Daily foot inspections	428 (60.8%)
Regular use of moisturizer	278 (39.5%)
Use of specialized footwear	204 (29.0%)
Regular visits to healthcare professionals	190 (27.0%)
Performing regular foot hygiene	532 (75.6%)

These findings indicate that although participants were aware of the importance of foot care, translating knowledge into practice was limited in several key areas.

A chi-square test was performed to identify associations between knowledge scores (categorized as good knowledge: ≥7; poor knowledge: <7) and demographic variables like age, gender, education level, and duration of diabetes. Education level significantly influenced knowledge scores (p<0.001). Participants with higher education (secondary and above) were more likely to have good knowledge (good knowledge in 190/266; 71.4%). No significant association was found between knowledge scores and gender (p=0.08).

**Table 5** depicts the logistic regression analysis identifying predictors of good foot care practices. It shows that higher knowledge (OR: 2.4) and positive attitudes (OR: 1.7) were significant predictors, emphasizing the importance of educational programs in fostering better practices.

**Table 5:** Knowledge and demographic variables.

Variable	Good knowledge, n(%)	Poor knowledge, n(%)	p-value
Age Group			
<40 years	89 (67.4%)	43 (32.6%)	0.03
40–60 years	298 (71.6%)	118 (28.4%)	
>60 years	83 (53.2%)	73 (46.8%)	
Education Level			
No formal education	32 (32.7%)	66 (67.3%)	<0.001
Primary education	116 (56.9%)	88 (43.1%)	
Secondary education	190 (71.4%)	76 (28.6%)	
Higher education	110 (80.9%)	26 (19.1%)	

Higher knowledge scores were associated with higher attitude scores.

**Table 6** depicts the influence of education level on practice scores. Participants with higher education demonstrated significantly better practices, with a mean practice score of 5.3 ± 0.9. This underscores the need for tailored interventions for individuals with lower education levels.

**Table 6:** Correlation between knowledge and attitude scores.

Variable	Mean ± SD	r-value	p-value	Interpretation
Knowledge Scores	6.5 ± 1.8	0.45	<0.001	Moderate positive correlation
Attitude Scores	3.8 ± 1.2			

**Table 7** depicts the logistic regression analysis of predictors for good foot care practices. It shows that participants with good knowledge were 2.4 times more likely to engage in good practices (p<0.001). Similarly, a positive attitude increased the likelihood of adopting better practices (OR: 1.7, p=0.002). Longer diabetes duration (>10 years) was also associated with improved practices (OR: 1.9, p=0.01), suggesting that sustained exposure to diabetes management positively impacts behavior.

**Table 7:** Logistic regression predictors of good practices.

Predictor	Odds ratio (OR)	95% CI	p-value	Interpretation
Good Knowledge	2.4	1.8–3.2	<0.001	Knowledgeable participants were more likely to engage in good practices.
Positive Attitude	1.7	1.3–2.4	0.002	Positive attitude increased likelihood of good practices.
Duration of Diabetes >10 years	1.9	1.2–3.0	0.01	Longer diabetes duration linked to better practices.

**Table 8** shows the practice scores categorized by education level, revealing a significant difference (F=8.12, p <0.001). Participants with higher education achieved the best practice scores (mean score 5.3 ± 0.9), while those without formal education had the lowest scores (mean score 3.2 ± 1.4). These findings underscore the critical role of education in improving adherence to foot care practices.

**Table 8:** Practice scores by education level (ANOVA Results).

Education level	Mean practice score ± SD	F-value	p-value	Interpretation
No Formal Education	3.2 ± 1.4	8.12	<0.001	Education level significantly impacted practice scores.
Primary Education	4.1 ± 1.2			
Secondary Education	4.8 ± 1.0			
Higher Education	5.3 ± 0.9			

Participants with higher education levels showed significantly better practice scores.

## Discussion

The findings of this study highlight several important observations related to diabetic foot care among patients attending a tertiary care hospital. Although participants demonstrated a moderate level of knowledge, critical gaps remain, particularly in translating this knowledge into consistent preventive practices. This is consistent with findings from earlier studies, including Monteiro-Soares et al. and Lavery et al., which underscore the role of awareness and education in promoting foot care behaviors among diabetic patients (11, 17, 18).

The amount of education proved to be a crucial predictor of sound knowledge and behaviors. Individuals with more excellent education had superior knowledge, with 80.9% demonstrating good knowledge compared to 32.7% lacking formal education (p < 0.001). This corresponds with a 2023 study from India, which indicated a significant link between educational achievement and foot care practice (19). The findings suggest that educational interventions aimed at low-literacy individuals may significantly enhance foot care outcomes.

Attitudes significantly influenced foot care actions. Although 75.6% of participants recognized the need for foot care in diabetes control, only 35.8% demonstrated confidence in detecting foot problems. The absence of trust may impede proactive foot care practices, requiring focused educational initiatives to improve patients’ self-efficacy (20). A 2020 RCT meta analysis meta-analysis highlighted the significance of motivational interviewing and behavioral therapy in enhancing adherence to diabetic self-management, particularly foot care (21).

The study revealed a moderate positive correlation (r = 0.45, p < 0.001) between knowledge and attitude ratings, indicating that enhancing information may positively affect attitudes. This corresponds with the Health Belief Model, which asserts that individuals with greater comprehension and perceived advantages are more inclined to engage in preventative behaviors (22). Future interventions ought to incorporate these theoretical frameworks to develop effective educational programs and improve long-term foot care outcomes.

Research findings indicated substantial deficiencies in standard foot care practices. For example, merely 27% of participants indicated consistent consultations with healthcare specialists for foot care. The limited utilization of professional services may be ascribed to cost, accessibility, and cultural beliefs (23). A 2022 study in Sub-Saharan Africa underscored comparable difficulties, stressing the necessity for community-based podiatry services to enhance access and affordability (24). Additionally, studies by Ferreira L et al. (20) and Najafi et al. (25) have highlighted the importance of integrating wearable technology and digital tools in improving adherence to foot care practices, particularly in low-resource settings (20, 25).

The research also revealed substantial correlations between the length of diabetes and foot care practices. Individuals with diabetes for over a decade exhibited a higher propensity for adopting favorable practices (OR: 1.9, p = 0.01). This research indicates that prolonged exposure to diabetes care may enhance awareness and implementation of preventive strategies. Nonetheless, it emphasizes the necessity of early education to avert difficulties in newly diagnosed patients (26, 27).

Recent studies further support these findings. Xu et al. (28) demonstrated that health education significantly improved both knowledge and self-care behaviors among diabetic patients (28). Pourkazemi et al. (29) similarly found that while many patients had moderate knowledge levels, actual practices were inadequate, indicating a need for better educational strategies (29). Sayampanathan et al. (30) explored patient-reported barriers and highlighted the enabling role of structured education programs, particularly in community-based settings (30).

The findings advocate for a comprehensive strategy in diabetes management that emphasizes foot care education. Healthcare practitioners want to integrate regular foot care evaluations and guidance into diabetes management strategies. Moreover, community outreach programs and telemedicine initiatives could improve access to podiatric services, especially in underserved regions (25).

While the cross-sectional nature of this study limits the ability to draw causal inferences, it provides a valuable snapshot of foot care knowledge, attitudes, and practices among diabetic patients in a real-world clinical setting. The use of univariate regression analysis, though informative in identifying initial associations, does not fully adjust for potential confounders; future research incorporating multivariate models could yield deeper insights. Additionally, although the study was conducted in a single tertiary care hospital using convenience sampling, the relatively large and diverse sample of 704 participants offers meaningful insights into the challenges and opportunities for improving diabetic foot care, especially in similar healthcare settings. These findings can serve as a foundation for broader, multi-center studies and targeted interventions.

## Conclusion

The findings underscore the need for tailored interventions, particularly for low-literacy and newly diagnosed patients, to enhance foot care awareness and adherence. Community-based podiatric services and telemedicine could address accessibility challenges, ensuring broader reach. By addressing these gaps, healthcare systems can significantly reduce the burden of diabetic foot complications and improve patient outcomes. Future research should focus on longitudinal studies and the evaluation of intervention programs to establish sustainable improvements in diabetic foot care.

## Data Availability

The raw data supporting the conclusions of this article will be made available by the authors, without undue reservation.
